# Development and Validation of a Risk Prediction Model for Breast Cancer Prognosis Based on Depression-Related Genes

**DOI:** 10.3389/fonc.2022.879563

**Published:** 2022-05-10

**Authors:** Xuan Wang, Neng Wang, Linda L. D. Zhong, Kexin Su, Shengqi Wang, Yifeng Zheng, Bowen Yang, Juping Zhang, Bo Pan, Wei Yang, Zhiyu Wang

**Affiliations:** ^1^ State Key Laboratory of Dampness Syndrome of Chinese Medicine, The Second Affiliated Hospital of Guangzhou University of Chinese Medicine, Guangzhou, China; ^2^ The Research Center of Integrative Cancer Medicine, Discipline of Integrated Chinese and Western Medicine, the Second Clinical College of Guangzhou University of Chinese Medicine, Guangzhou, China; ^3^ The Research Center for Integrative Medicine, School of Basic Medical Sciences, Guangzhou University of Chinese Medicine, Guangzhou, China; ^4^ Guangdong-Hong Kong-Macau Joint Lab on Chinese Medicine and Immune Disease Research, Guangzhou University of Chinese Medicine, Guangzhou, China; ^5^ School of Chinese Medicine, Hong Kong Baptist University, Hong Kong, Hong Kong SAR, China; ^6^ School of Pharmaceutical Sciences, Guangzhou University of Chinese Medicine, Guangzhou, China; ^7^ Guangdong Provincial Key Laboratory of Clinical Research on Traditional Chinese Medicine Syndrome, Guangdong Provincial Academy of Chinese Medical Sciences, Guangdong Provincial Hospital of Chinese Medicine, Guangzhou, China; ^8^ Atrius Health, Harvard Vanguard Medical Associates, Burlington, MA, United States

**Keywords:** breast cancer, depression, predictive model, overall survival, nomogram

## Abstract

**Background:**

Depression plays a significant role in mediating breast cancer recurrence and metastasis. However, a precise risk model is lacking to evaluate the potential impact of depression on breast cancer prognosis. In this study, we established a depression-related gene (DRG) signature that can predict overall survival (OS) and elucidate its correlation with pathological parameters and sensitivity to therapy in breast cancer.

**Methods:**

The model training and validation assays were based on the analyses of 1,096 patients from The Cancer Genome Atlas (TCGA) database and 2,969 patients from GSE96058. A risk signature was established through univariate and multivariate Cox regression analyses.

**Results:**

Ten DRGs were determined to construct the risk signature. Multivariate analysis revealed that the signature was an independent prognostic factor for OS. Receiver operating characteristic (ROC) curves indicated good performance of the model in predicting 1-, 3-, and 5-year OS, particularly for patients with triple-negative breast cancer (TNBC). In the high-risk group, the proportion of immunosuppressive cells, including M0 macrophages, M2 macrophages, and neutrophils, was higher than that in the low-risk group. Furthermore, low-risk patients responded better to chemotherapy and endocrine therapy. Finally, a nomogram integrating risk score, age, tumor-node-metastasis (TNM) stage, and molecular subtypes were established, and it showed good agreement between the predicted and observed OS.

**Conclusion:**

The 10-gene risk model not only highlights the significance of depression in breast cancer prognosis but also provides a novel gene-testing tool to better prevent the potential adverse impact of depression on breast cancer prognosis.

## Introduction

As a formidable health problem for women worldwide, breast cancer ranks first in incidence and mortality among women’s malignancies. According to the World Health Statistics 2020, breast cancer incidence accounted for 11.7% of new cases in 2020 and 6.9% of mortalities worldwide (1). Despite advances in early detection and drug development, significant barriers remain in the prediction of metastasis or recurrence risk (2). Given the long latent period and relatively young age of breast cancer, the development of prognosis-risk prediction models is of great value to improve treatment strategies and overall survival (OS).

Prognostic risk factors of breast cancer have attracted increasing interest for more than a decade. In 2007, the St. Gallen Expert Consensus considered that the risk of breast cancer recurrence was mainly related to age, tumor size, histological grade, peritumor intravascular cancer emboli, and expression of the estrogen receptor (ER)/progesterone receptor (PR) and human epidermal growth factor receptor 2 (HER2) (3). In St. Gallen 2011, breast cancer was divided into four types, namely, luminal A, luminal B, HER2+, and triple-negative breast cancer (TNBC) (4), of which TNBC was considered to have a poor prognosis. In fact, only 77% of TNBC patients survive 5 years after diagnosis, while the survival rate of other subtypes reached 93% (5). The 5-year survival rate in the metastatic TNBC is less than 30% and almost all patients will ultimately die of their disease (6). However, breast cancer is a highly heterogenous disease (7), and some TNBC patients have a low recurrence risk and relatively long survival period. It is believed that more genes are involved in determining breast cancer prognosis in addition to ER, PR and HER2. For example, the luminal A type was further classified into low-, intermediate-, and high-risk recurrence subgroups by a 21-gene recurrence score system (8). In addition, risk prediction tools, such as MammaPrint, Breast Cancer Index, PAM50 and EPclin, have also been developed to address the needs for precise diagnosis and treatment (9–12). Nevertheless, these tools are greatly limited by specific platforms and pathological subtype. Thus, it is significant and urgent to establish novel gene panels to assist with risk prediction and anti-cancer drug development.

Numerous studies have emphasized the close correlation between depression and breast cancer (13–16). The meta-analysis showed that 32.3% of breast cancer patients suffer from depression (17). Nearly half of breast cancer patients at early stage would have depression, anxiety, or both after diagnosis (18). Depression is related to a 24% increased risk of breast cancer recurrence, 30% increased risk of all-cause mortality, as well as 29% increased risk of cancer-specific mortality (19). Animal studies have demonstrated that psychological stress could accelerate tumor growth and promote lung metastasis (20–22). Thus, it is crucial to explore the role of depression-related gene (DRGs) for the prognosis of breast cancer.

Herein, a 10-DRG risk signature was determined and it presented a positive prediction with OS in The Cancer Genome Atlas (TCGA) and GSE96058 cohorts, particularly for TNBC patients. A high-risk score was related to age, tumor size, tumor stage, metastasis, and immunosuppressive cells. Moreover, low-risk patients might be highly sensitive to chemotherapy and endocrine therapy. Finally, a nomogram was successfully established by integrating the risk score and other clinical parameters. Taken together, our study not only provides a novel risk prediction model for evaluating breast cancer prognosis based on DRGs analysis but also highlights the clinical significance of depression evaluation in breast cancer treatment.

## Materials and Methods

### Data Source and Collection

Breast cancer related clinical information and the gene expression profiles were collected from TCGA database, which was chosen as testing dataset containing 1,096 patient samples and 113 non-tumor tissues. For further verification, the dataset GSE96058 (n=2969) downloaded from the GEO database was selected for validation.

### Selection of DEGs in Breast Cancer

A total of 8,479 DRGs were acquired from the Genecard database, which supplies a comprehensive, up-to-date list of human genes. We obtained the expression values of 8,479 DRGs from TCGA. Based on TCGA dataset, DEGs were identified *via* Wilcoxon test after averaging replicate probes. Those genes with |log2-fold change (FC)| > 2 and adjusted *P* value (FDR) < 0.05 were considered DEGs.

Subsequently, PPIs of the DEGs were constructed based on the STRING (The Search Tool for the Retrieval of Interacting Genes) online tool. Then Cytoscape software 3.6.1 was applied to visualized the network. Additionally, the key modules in network were analyzed by MCODE in Cytoscape software. The options were set as degree cutoff = 2, K-Core = 4, and Node Score Cutoff = 0.3.

### Identification of Prognostic Genes and Construction of a Risk Model

The univariate Cox regression analysis was performed to explore the correlation between genes and OS in the testing dataset. DEGs with *p <*0.05 were considered as candidate genes. Then LASSO regression was applied to shrink scope of gene screening. Finally, a multivariate Cox analysis was constructed to screen highly prognosis-associated genes and generate the prognostic risk model.

The risk score was calculated as follows:


$$\text{Risk Score }(\text{patient})=\sum_{i=1}^{n}\beta \text{i}Exp\text{i}.$$

(where “*Exp*” represents gene expression level and “β” represents the regression coefficient from the multivariate Cox analysis).

According to the risk score, the optimal cut-off value obtained by Receiver operating characteristic (ROC) analysis was performed for patient risk stratification.

### Validation of the Constructed Model

To validate this model, Kaplan-Meier survival analysis along with log-rank test was employed to analyze survival difference between patients with different scores. To confirm whether the signature can be applied as an independent factor predictive of survival, univariate and multivariate Cox regression analyses were carried out.

### GSEA

To explore the potential biological pathways, GSEA was carried out during the enrichment of the MSigDB Collection (c2.cp.kegg.v7.1.symbols.gmt). Pathways with |NES| > 1 and *p* < 0.05 were considered significantly enriched.

### Exploration of Tumor-Infiltrating Immune Cells

As the most frequently cited tool for analyzing immune cells infiltration, CIBERSORT was used to quantify the immune cell proportion based on their RNA sequencing data (23). In the study, the tool was used to estimate the proportion of immune cell fractions in each breast patients in TCGA cohort.

### Investigation of the Significance of Risk Signature for Drug Treatment

Genomics of Drug Sensitivity in Cancer website (24) was employed to discuss differences between patients with low and high score in response to chemotherapy, endocrine therapy, and targeted therapy. The IC_50_ of common therapeutic drugs in the TCGA breast cancer cohort was calculated.

### Identification of Potential Small Molecule Drugs Targeting High Risk Patients

CMap is an online pharmacogenomic database based on gene expression data of cultured human cells treated with bioactive substance. Those up- and down-regulated target genes were uploaded to CMap. The connectivity score ranges from -1 to 1, which was used to reveal the closeness between expression profiles. The drugs with negative scores were generally considered as potential therapeutic molecules.

### Construction and Evaluation of the Nomogram

The rms R package was performed to build a nomogram containing the model and clinicopathological features. The C-index and calibration plot were applied to assess predicted probabilities.

### Statistical Analysis

The R software (version 3.6.3) was used to analyze the statistical data. Kaplan-Meier survival curves at 1-, 3-, and 5-years were illustrated with the survminer package. The prognostic effect of the risk model and clinicopathological features were assessed through univariate Cox analysis and multivariate Cox analysis. A two-sided *p* < 0.05 was considered statistically significant.

## Results

### Identification of DRGs and Establishment of a 10-Gene Risk Model for Predicting Breast Cancer Prognosis

**Figure 1:**
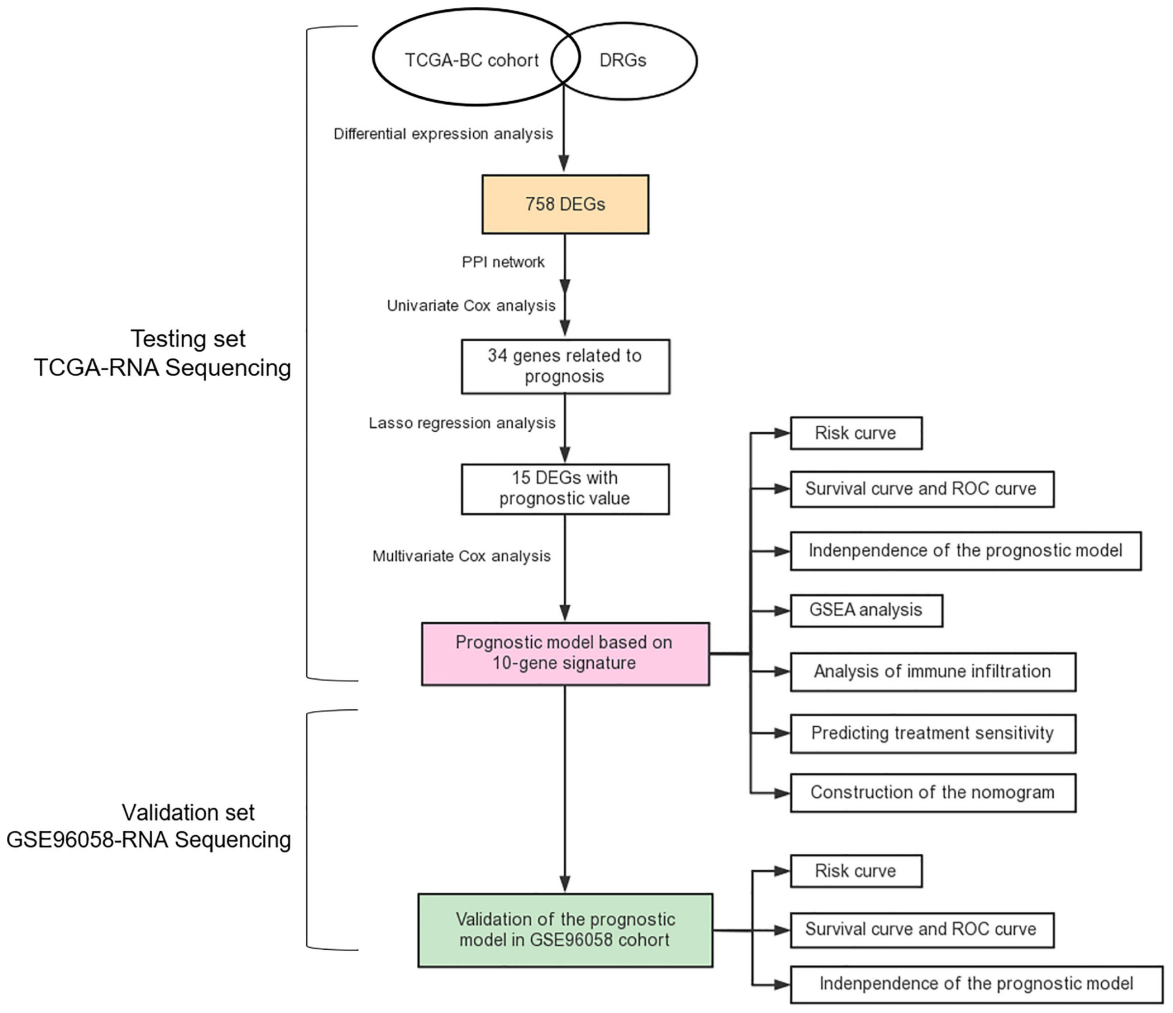
Flow chart of this study.

**Figure 2:**
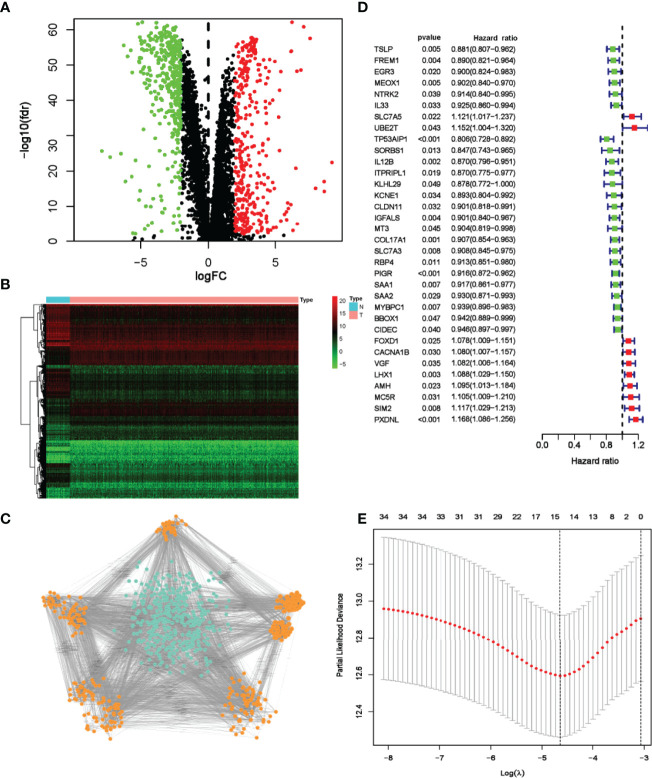
Screening and identification of prognostic DRGs of TCGA breast cancer patients. **(A)** Volcano map visualizes DEGs between breast cancer and normal tissues. Red dots indicate upregulated genes, green dots indicate downregulated genes, and black dots indicate no differences gene; **(B)** Heatmap analysis of differential DRGs; **(C)** MCODE analysis from the PPI network. The orange nodes represent genes in significant module, while the blue nodes represent genes in insignificant module. **(D)** Forest plot of 34 genes identified by the univariate Cox regression analysis. **(E)** LASSO coefficient profiles of 15 prognosis-related genes. The red dots represent the partial likelihood values. The optimal parameter (λ) was calculated by ten-fold cross-validation.

To construct a risk model based on DRGs, TCGA-BC cohort and GSE96058 cohort were selected as testing and validation datasets, respectively. The flow chart of bioinformatic analysis is illustrated in **Figure 1**. This study included 1,027 cases from TCGA cohort in which primary breast cancer had been followed up for more than 1 month. Clinical characteristics are summarized in **Table S1**. In total, 358 upregulated and 400 downregulated DRGs were finally identified (**Figures 2A, B**). To narrow the gene set and determine the hub genes, a protein–protein interaction (PPI) network based on the differentially expressed genes (DEGs) was built. Subsequently, module analyses of the network were conducted using Molecular Complex Detection (MCODE) to obtain hub genes (**Figure 2C**). Five significant modules were screened out and genes identified as hub genes were further analyzed.

In univariate Cox analysis, 34 DRGs were found significantly related to OS (**Figure 2D**). Through lasso regression analyses, 15 genes were further identified as prognosis-related genes (**Figure 2E**). Multivariate Cox analysis confirmed the following 10 DRGs for constructing the risk model: *MT3*, *SORBS1*, *IGFALS*, *AMH*, *IL12B*, *TP53AIP1*, *PXDNL*, *MC5R*, *FOXD1* and *LHX1* (**Table S2**). The formula was developed as below:


$$\begin{array}{l} \text{Risk score}=(-0.160)\times \text{Ex}{\text{p}}_{\left(MT3\right)}+(-0.129)\\ \;\times \text{Ex}{\text{p}}_{\left(SORBS1\right)}+(-0.084)\times \text{Ex}{\text{p}}_{\left(IGFALS\right)}+0.089\\ \;\times \text{Ex}{\text{p}}_{\left(AMH\right)}+(-0.137)\times \text{Ex}{\text{p}}_{\left(IL1 2B\right)}\\ \;+(-0.164)\times \text{Ex}{\text{p}}_{\left(TP5 3AIP1\right)}+0.137\times \text{Ex}{\text{p}}_{\left(PXDNL\right)}\\ \;+0.120\times \text{Ex}{\text{p}}_{\left(MC5R\right)}+0.073\times \text{Ex}{\text{p}}_{\left(FOXD1\right)}\\ \;+0.055\times \text{Ex}{\text{p}}_{(LHX1).} \end{array}$$

### Prognostic Effect of the Model on Predicting OS of Breast Cancer

**Figure 3:**
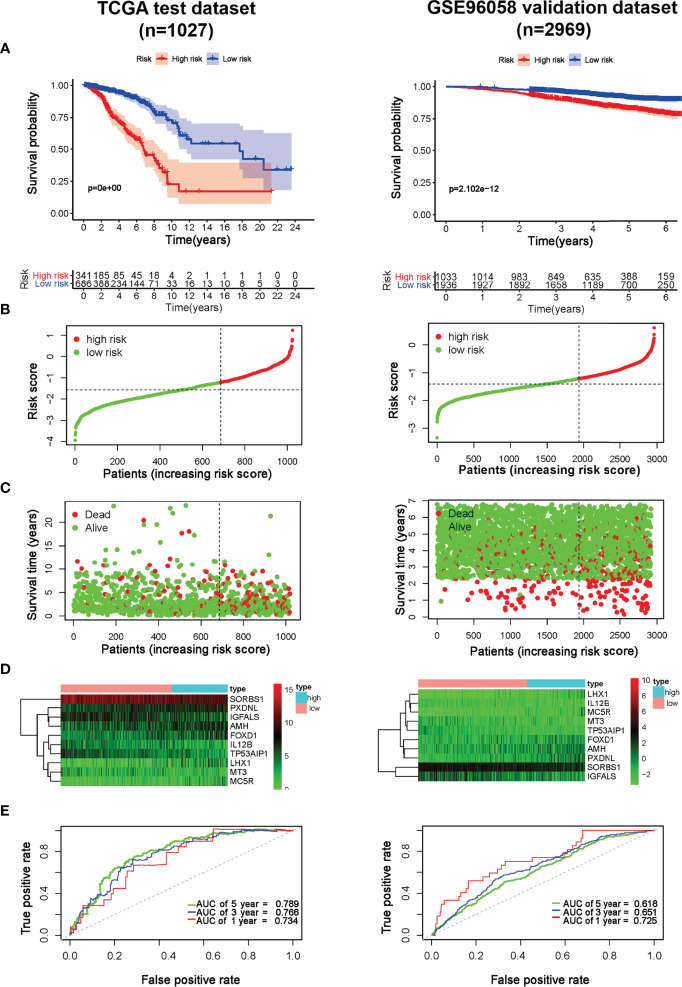
Prognostic effect of the model in breast cancer. **(A)** Kaplan-Meier OS curves of breast cancer patients in TCGA and GSE96058 cohorts; **(B)** The distribution of risk score of breast cancer patients in TCGA and GSE96058 cohorts. X‐axis here is patient number and Y‐axis is risk score; **(C)** Survival status of the patients with different risk score in TCGA and GSE96058 cohorts. X‐axis here is patient number and Y‐axis is survival time; **(D)** A heatmap showing 10 gene expression profiles in TCGA and GSE96058 cohorts; **(E)** ROC Analysis of the risk model in TCGA and GSE96058 cohorts.

To explore the predictive effect of the model, risk scores in TCGA-Breast cancer and GSE96058 cohorts were calculated according to the formula. Subsequently, patients were divided into either low- or high-risk group based on the cut-off point of −1.281, as determined by MedCalc version 15.2.2 (MedCalc Software bvba, Ostend, Belgium). Survival analysis demonstrated a shorter survival time in high-risk patients (*p* < 0.001), which was verified by the GSE96058 dataset (*p* < 0.001) (**Figure 3A**). As illustrated in the **Figures 3B, C**, the higher the score, the higher proportion of patients dying in TCGA and GSE96058 datasets. Heatmap analysis showed that *MT3*, *SORBS1*, *IGFALS*, *IL12B* and *TP53AIP1* in the low-risk group were overexpressed, whereas *AMH*, *PXDNL*, *MC5R*, *FOXD1* and *LHX1* in the high-risk group were overexpressed. The outcomes in GSE96058 dataset were in line with those in TCGA (**Figure 3D**). As displayed in **Figure 3E**, the maximum area under the ROC curve (AUC) values reached 0.789, indicating the good sensitivity and specificity of the model. Consistently, in GSE96058 cohort the model achieved the maximum AUC of 0.725. These results revealed that the model was effective in predicting the survival probability.

Considering tumor heterogeneity, survival and ROC analyses of different molecular subtypes of breast cancer were carried out, respectively. The risk model had good prognostic performance, particularly for TNBC. As shown in **Figure S1**, the survival analyses proved the prognostic value for TNBC in both TCGA and GSE96058 cohorts (*p* < 0.001). ROC analyses further revealed that in the TCGA cohort, the AUC of TNBC was 0.709, 0.732, and 0.730 at 1, 3 and 5 years, respectively. The results were validated in the GSE96058 dataset in which AUC at 1, 3, and 5 years reached 0.747, 0.657, and 0.609 for TNBC, respectively (**Figure S2**).

### Correlation Analysis Between the Model and Pathological Parameters

**Figure 4:**
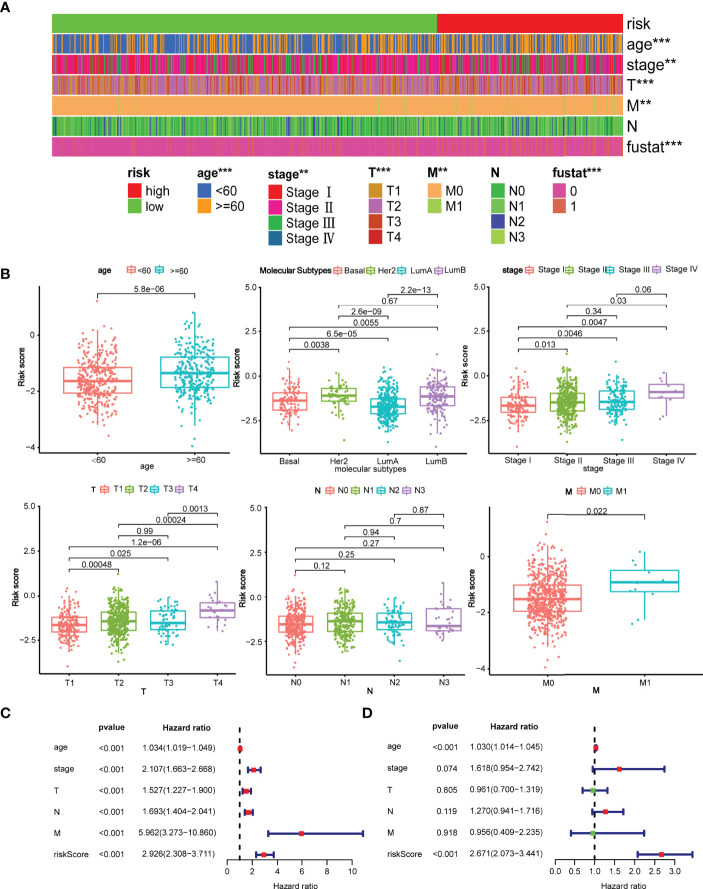
Clinical evaluation of the risk model with pathological parameters. **(A)** The distribution of the model with the clinicopathological features including age, stage, TNM stage, survival state; **p < 0.01 and ***p < 0.001. **(B)** Comparison of risk score between patients with different pathological parameters. Age, molecular subtype, clinical stage, T stage, M stage were significantly associated with the risk score, while N stage was not; **(C)** Forrest plot of univariate Cox regression analysis. The result revealed that age, clinical stage, T stage, N stage, M stage, and risk score were statistically different. **(D)** Forrest plot of multivariate Cox regression analysis. Either age or risk score acted as an independent prognostic factor.

The relationship between the risk model and pathological parameters was further investigated. As displayed in **Figures 4A, B**, the risk model in the TCGA testing cohort was closely associated with age, molecular subtypes, clinical stage, T stage, and M stage, whereas N stage was not. Similarly, significant differences were also observed in GSE96058 cohorts, including age, molecular subtypes, tumor size, and positive nodes (**Figure S3A, B**). The risk score was significant for prognosis prediction when evaluated by univariate Cox analysis (HR = 2.926, 95% CI: 2.306–3.711) (**Figure 4C**). Furthermore, multivariable Cox analysis revealed that the model remained statistically significant (HR = 1.075, 95% CI:1.058–1.092), indicating that the model is an independent prognostic factor (**Figure 4D**). Likewise, in GSE96058 cohort, the univariate Cox regression analysis demonstrated that risk score (HR=2.329, 95% CI:1.879–2.887) is significantly related to survival (**Figure S3C**). The multivariate Cox regression analysis indicated that risk score (HR=1.648, 95% CI:1.324–2.049) is still an independent prognostic factor for breast cancer (**Figure S3D**).

### Molecular Mechanism Analysis of the Model in Predicting Prognosis

**Figure 5:**
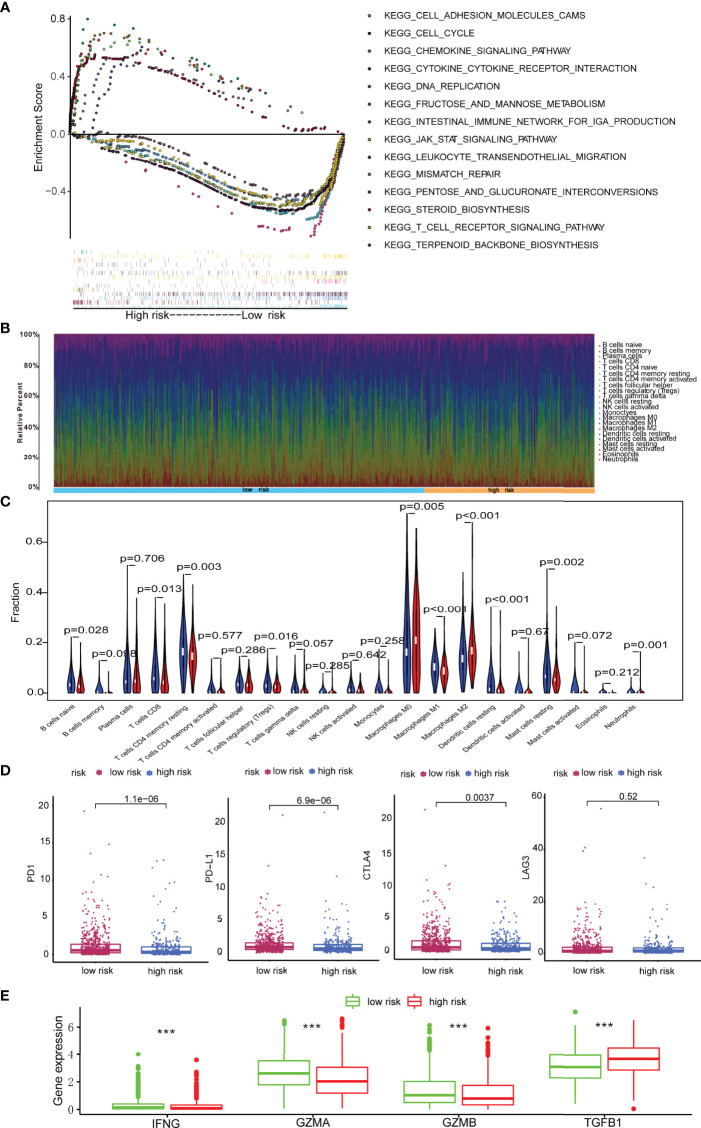
Immune landscape in breast cancer patients with low- and high-score. **(A)** GSEA revealing biological processes correlated with risk model; **(B)** The abundances of immune cells in tumor microenvironment; **(C)** Comparison of proportion of immune cells types in patients with low- and high-score; **(D)** Immune checkpoints including PD1, PD-L1, CTLA4, and LAG3 expression in patients with low-score and high-score. **(E)** Expression levels of IFNG, GZMA, GZMB and TGFB1 in patients with low-score and high-score. ***p < 0.001.

We continued to explore the potential biological mechanisms of the risk model. GSEA revealed that group at high-risk was enriched in terpenoid backbone biosynthesis, cell cycle, pentose, and glucuronate interconversions, DNA replication, steroid biosynthesis, fructose, and mannose metabolism as well as mismatch repair. By contrast, the group at low-risk was enriched in pathways such as cell adhesion molecules, intestinal immune network for IgA production, cytokine-cytokine receptor interaction, JAK-STAT signaling pathway, chemokine signaling pathway, T cell receptor signaling pathway, and leukocyte transendothelial migration (**Figure 5A**).

Growing evidence shows that depression could inhibit the immune effector cells and facilitate immune escape, thus protecting cancer cells from immunological killing (14, 25). Moreover, the preliminary GSEA analyses in this study illustrated that the signature is closely associated with immune pathways. Herein, the abundance of 22 immune cells in low-risk and high-risk cases was analyzed *via* the CIBERSORT method (**Figure 5B**). The results revealed that high-risk patients had higher abundances of immunosuppressive cells including M0 macrophages, M2 macrophages, and neutrophils, but significantly lower abundances of M1 macrophages, naive B cells, CD8 T cells, resting memory CD4 T cells, regulatory T cells, resting dendritic cells, and resting mast cells (**Figure 5C**). In summary, the immunosuppressive microenvironment might be responsible for worse prognosis in high-risk cases.

Then the expression of the immune checkpoint regulators was analyzed in patients with different risk score. As shown in **Figure 5D**, the expressions of PD-1, PD-L1, and CTLA-4 increased significantly in patients with low-scores (*p* < 0.05), whereas LAG3 showed no statistical difference. These results implied that low-risk patients might be sensitive to immune checkpoint blockade therapies.

Additionally, it is well established that cytokines play a crucial role in the tumor immune microenvironment. Therefore, the results in TCGA cohort demonstrated that GZMA, GZMB, and IFNG in the low-risk group were highly expressed, while TGFB1 was elevated in high-risk breast cancer patients (p < 0.001) (**Figure 5E**).

### Clinical Effects of the Model in Predicting Treatment Sensitivity

**Figure 6:**
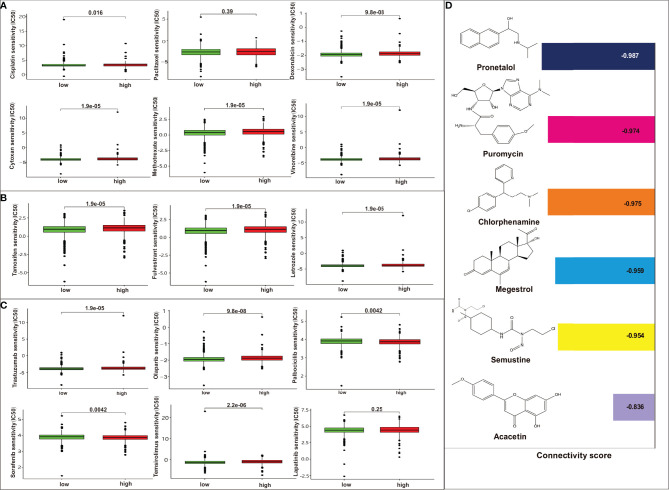
Analysis of the association between the risk model and chemotherapeutics, endocrine therapy, and targeted therapy. **(A)** The model predicting the sensitivity to chemosensitivity. It was estimated that low-risk patients had lower IC_50_ for chemotherapeutics such as paclitaxel, cisplatin, and doxorubicin, cytoxan, methotrexate, vinorelbine, except for paclitaxel; **(B)** The model predicting the sensitivity to endocrine therapy. It was estimated that low-risk patients had lower IC_50_ such as tamoxifen, fulvestrant, and letrozole. **(C)** The model predicting the sensitivity to targeted therapy. It was estimated that low-risk patients had lower IC_50_ such as trastuzumab, olaparib, temsirolimus, whereas high-risk patients had lower IC_50_ for targeted therapy such as palbociclib, sorafenib. There are no differences between high risk or low risk patient for lapatinib; **(D)** CMap analysis for high-risk score patients. Six targeted drugs such as pronetalol, puromycin, chlorphenamine, megestrol, semustine, and acacetin are predicted therapy for this risk score in breast cancer.

We further identified associations between the model and treatment effect of chemotherapeutics, endocrine drugs, and targeted agents. Low-risk patients had lower half-maximal inhibitory concentration (IC_50_) of chemotherapeutics such as cisplatin, doxorubicin, cytoxan, methotrexate, and vinorelbine except for paclitaxel, indicating the predictive potential of the model for chemosensitivity (**Figure 6A**). Regarding endocrine therapy, low-risk patients had lower IC_50_ of endocrine therapies, such as tamoxifen, fulvestrant, and letrozole (**Figure 6B**). Low-risk patients had lower IC_50_ of targeted therapies, such as trastuzumab, olaparib, and temsirolimus, whereas high-risk patients had lower IC_50_ for targeted therapies such as palbociclib and sorafenib. The two groups showed no statistical differences for lapatinib (**Figure 6C**). Moreover, the DEGs were subjected to Connectivity Map (CMap) analysis and a total of six drugs, including pronetalol, puromycin, chlorphenamine, megestrol, semustine, and acacetin, were indicated for high-risk breast patients (**Figure 6D**).

### Nomogram Construction Based on the 10-Gene Risk Model

**Figure 7:**
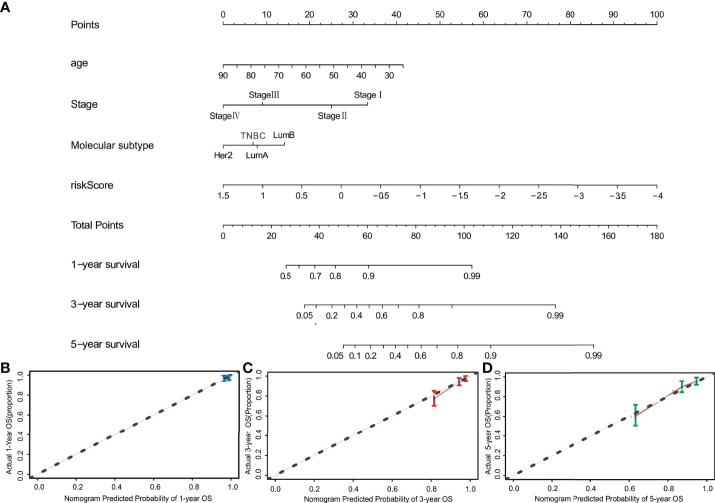
Construction of the nomogram. **(A)** The nomogram for predicting 1-, 3-, and 5-year OS was constructed by combining risk score, age, stage, and molecular subtype. **(B–D)** The calibration curves of nomograms between predicted and observed 1-, 3- and 5-year OS. The dashed line of 45° indicates the perfect prediction.

To create a quantitative method to predict OS, the nomogram for predicting 1-year, 3-year and 5-year OS was developed. Four risk factors, including age, stage, molecular subtypes, and risk score, were included in the nomogram (**Figure 7A**). The C-index reached 0.782 (95%CI: 0.733–0.831). As shown by the calibration curves, the actual and predicted OS matched well (**Figures 7B–D**).

## Discussion

In this study, a novel prognostic model consisting of 10-DRGs was established to enhance predictive capability of OS in breast cancer. The findings demonstrated that the model can effectively distinguish high-risk from low-risk patients and the model was effective in predicting OS in both the testing and validation sets, particularly for TNBC patients. Additionally, when pathological factors were included in multivariate Cox analysis, the model remained as an independent prognostic factor. These results implied that the risk model is a robust prognostic tool. Moreover, it presented that a high score was related to poor survival in TNBC patients. Given that TNBC is the most challenging subtype of breast cancer, predicting prognosis of TNBC by this risk model is of great significance.

Among the 10 genes, five (*PXDNL*, *MC5R*, *AMH*, *FOXD1*, and *LHX1*) were high-risk genes and five (*IGFALS*, *SORBS1*, *IL12B*, *MT3*, and *TP53AIP1*) were protective factors. *PXDNL* is reported as a susceptibility gene in patients with depression (26). *MC5R*, a melanocortin receptor, could mediate the axis responsiveness to integrated signals from diurnal rhythms and cortisol negative feedback. It was reported that MC5R antagonist could treat the depressive or generalized anxiety disorder (27). *MT3* may be related to the antidepressant-like activity of eugenol, resulting in increased expression of the brain-derived neurotrophic factor (28). Furthermore, compared with those of the wild-type mice, Koumura et al. (29) found that *MT3* KO mice had shorter social interactions duration as well as diminished prepulse inhibition for the acoustic startle response, indicating abnormal psychological behavior in schizophrenia, anxiety, autism, and phencyclidine-induced psychosis. In general, the 10 genes are related to depression but are not solely related to depression. The mental diseases including depression, anxiety, and panic are usually interrelated. Patients with depression, anxiety, or panic experienced declines in quality of life and showed poor treatment adherence as compared with patients without mental disorders (30). Our previous meta-analysis indicated that depression and anxiety would increase the risk of breast cancer recurrence and mortality (19). Likewise, the 10 genes included in this study are not only depression-related but also related to breast cancer progression. Gomulkiewicz et al. (31) demonstrated that patients with ductal breast cancer had lower expression of *MT3* than that in non-malignant breast tissue and the level of *MT3* in breast cancer patients with lymph node metastasis decreased compared to patients without metastasis. Moreover, the expression of *MT3* in breast cancer cell lines was lower than that in the normal human breast epithelial cell lines. These findings indicated that *MT3* might be correlated with the malignant transformation of breast epithelial cells and tumor progression. In addition, *PXDNL* and *FOXD1* were reportedly involved in breast cancer pathogenesis and were significant in predicting prognosis. FOXD1 was found to be significantly related to the prognosis of basal-like breast cancer (32). Zhao et al. (33) demonstrated that *FOXD1* could increase cell proliferation and enhance chemoresistance of cancer cells by targeting p27. It was demonstrated that decreased levels of *SORBS1* have significant correlation with worse survival, distant metastasis, and more malignant phenotype. *SORBS1* could inhibit tumorigenesis and metastasis through preventing JNK activation and attenuate cisplatin chemotherapy through p53 accumulation in breast cancer (34). *TP53AIP1* inhibits proliferation and growth of breast cancer cells through cell-cycle arrest, apoptosis induction, elevation of the expression of cleaved-caspase-3, cleaved-caspase-9, Bax, p53, and the inhibition of Bcl-2, Ki67, and PI3K/Akt pathways (35). Although reports on the prognostic effect of the remaining genes in breast cancer are limited, it is worth further evaluating their potential as biomarkers of breast cancer progression.

Furthermore, the model was associated with tumor-infiltrating immune cells, as the abundance of M2 macrophages and neutrophils in high-risk patients significantly increased. The abundance of M1 macrophages, naive B cells, CD8 T cells, resting memory CD4 T cells, resting dendritic cells, and resting mast cells was lower in the high-risk group and was related to better OS. It is well established that cytokines play a crucial role in the tumor immune microenvironment. As high-risk patients in our study had higher abundances of immunosuppressive cells, but significantly lower proportions of M1 macrophages, naive B cells, CD8 T cells, resting memory CD4 T cells, resting dendritic cells, resting mast cells, and immune inhibitory cytokines such as *TGFB1* and biomarkers of T cell activation such as *GZMA*, *GZMB*, and *IFNG* are selected to explore the differences between high-risk and low-risk groups. Notably, the results in TCGA cohort demonstrated that *GZMA*, *GZMB*, and *IFNG* in the low-risk cases were highly expressed, while *TGFB1* was elevated in the high-risk group. Future research should be conducted on the mechanisms of this model in influencing immune cells in tumor microenvironment. Accumulating evidence also demonstrated the potential of immune checkpoint inhibitors (ICIs) in breast cancer treatment (36); however, only a few patients obtained therapeutic benefit from ICIs. It was found that low-risk patients had increased expression of PD-1, PD-L1, as well as CTLA-4, suggesting that low-risk patients are promising candidates for ICIs. In addition, the model provided an opportunity to choose the optimal therapy for breast cancer patients. Our study revealed that low-risk patients seem to be more sensitive to chemotherapy and endocrine therapy, which is in line with better OS of the low-risk patients. These results indicate the potential effect of the model in predicting therapy sensitivity, which is also beneficial for selecting optimal therapeutic strategies and improving breast cancer patients’ prognosis.

Furthermore, our study provides potential therapeutic options for high-risk patients. Among them, pronethalol, a β-adrenoceptor antagonist, is used to treat coronary heart disease and arrhythmias. Clinical studies have demonstrated that the application of β-blocker could improve the prognosis of patients with lung cancer, ovarian cancer, and melanoma (37–39). Although the use of pronethalol in clinical practice was restricted because it proved to be a carcinogen in mice (40), other β-blocker agents, such as propranolol, may be a valid approach for patients at high risk. It was reported that propranolol inhibits metastasis, reduces recurrence, and cancer-specific mortality in several clinical studies (41, 42). In addition, the safety of propranolol in breast cancer patients has been proven in a phase II pilot study (43). Chlorpheniramine, an alkylamine antihistamine, is used to prevent allergic symptoms. Although not generally recognized as an antidepressant or anxiolytic drug, chlorphenamine seems to have these properties as well. Shoko et al. (44) found that chlorpheniramine exerted antidepressant-like effects by activating dopamine D1 and α1-adrenoceptors. This result indicates that antidepressants may be another choice for breast cancer patients. It was proved that application of antidepressants for breast cancer may reduce mortality risk (45). However, antidepressants, such as selective serotonin re-uptake inhibitor, can reduce tamoxifen’s effectiveness and increase death risk (46). Thus, further studies are warranted for validation. Acacetin is a natural flavonoid, which can inhibit the secretion of carcinogenic estrogen metabolites, as well as tumor cell proliferation, invasion, and migration (47, 48). It is worth exploring its potential application in regulating DRGs during breast cancer treatment.

## Conclusion

This study provides a novel 10-DRGs risk model to predict breast cancer survival. The model is particularly significant for TNBC and is promising for predicting drug sensitivity and is thus helpful for designing optimal therapeutic strategies to improve clinical prognosis. Our findings further highlight the significance of monitoring and treating psychological stress in preventing recurrence and metastasis of breast cancer. Further experimental validation and prospective clinical trials are worth conducting.

## Data Availability Statement

The original contributions presented in the study are included in the article/**Supplementary Material**. Further inquiries can be directed to the corresponding author.

## Author Contributions

ZW and XW conceived and designed the study. XW and NW conducted the bioinformatic analysis and wrote the manuscript. LZ, WY, and KS participated in data interpretation and revised the manuscript. SW, YZ, BY, JZ, and BP contributed to data collection and discussion.

## Funding

This work was supported by the National Natural Science Foundation of China [81873306, 81973526, 82004373, 82104869]; the State Key Laboratory of Dampness Syndrome of Chinese Medicine [SZ2021ZZ19]; Science and Technology Planning Project of Guangdong Province [2021A0505030059, 2017B030314166]; Department of Education of Guangdong Province [2018KZDXM022, A1-2606-19-111-009, 2019KQNCX019]; The 2020 Guangdong Provincial Science and Technology Innovation Strategy Special Fund (Guangdong-Hong Kong-Macau Joint Lab) [2020B1212030006]; Guangdong traditional Chinese medicine bureau project [20201132, 20211114]; Guangzhou science and technology project [202102010316, 202102010241, 201904010407]; The Specific Research Fund for TCM Science and Technology of Guangdong provincial Hospital of Chinese Medicine [YN2018MJ07, YN2018QJ08], and the Foundation for Young Scholars of Guangzhou University of Chinese Medicine [QNYC20190101].

## Conflict of Interest

The authors declare that the research was conducted in the absence of any commercial or financial relationships that could be construed as a potential conflict of interest.

## Publisher’s Note

All claims expressed in this article are solely those of the authors and do not necessarily represent those of their affiliated organizations, or those of the publisher, the editors and the reviewers. Any product that may be evaluated in this article, or claim that may be made by its manufacturer, is not guaranteed or endorsed by the publisher.
