# Supplementary material for: Development and Validation of a Risk Prediction Model for Breast Cancer Prognosis Based on Depression-Related Genes
Source: Front Oncol. 2022 May 10;12:879563. doi: 10.3389/fonc.2022.879563 (PMC9128552; doi:10.3389/fonc.2022.879563)
Supplement: Supplementary file 1 [file DataSheet_1.doc]

Supplementary Material

**Development and Validation of a Risk Prediction Model for Breast Cancer Prognosis Based on Depression-related genes**

**Table S1 | The clinical features of TCGA cohort and the GSE96058 cohort.**

|  | TCGA cohort | GSE96058 cohort |
| --- | --- | --- |
| **No. of patients** | 1027 | 2969 |
| **age** |  |  |
| < 60 | 553 | 1159 |
| >= 60 | 474 | 1910 |
| **Status** |  |  |
| Alive | 886 | 2747 |
| Dead | 141 | 322 |
| **Stage** |  |  |
| Stage I | 180 | - |
| Stage II | 579 | - |
| Stage III | 228 | - |
| Stage IV | 18 | - |
| Unknown | 22 | - |

**Table S2 Results of genes in 10-gene risk model via multivariable Cox analysis.**

| ID | coef | HR | HR.95L | HR.95H | P value |
| --- | --- | --- | --- | --- | --- |
| MT3 | -0.160 | 0.852 | 0.773 | 0.940 | 0.0014 |
| SORBS1 | -0.129 | 0.879 | 0.761 | 1.016 | 0.0802 |
| IGFALS | -0.084 | 0.919 | 0.854 | 0.990 | 0.0267 |
| AMH | 0.089 | 1.093 | 1.004 | 1.189 | 0.0392 |
| IL12B | -0.137 | 0.872 | 0.795 | 0.957 | 0.0039 |
| TP53AIP1 | -0.164 | 0.849 | 0.764 | 0.943 | 0.0022 |
| PXDNL | 0.137 | 1.146 | 1.065 | 1.234 | 0.0003 |
| MC5R | 0.120 | 1.127 | 1.028 | 1.236 | 0.0109 |
| FOXD1 | 0.073 | 1.076 | 1.004 | 1.152 | 0.0384 |
| LHX1 | 0.055 | 1.056 | 0.993 | 1.124 | 0.0836 |

HR: hazard ratio;


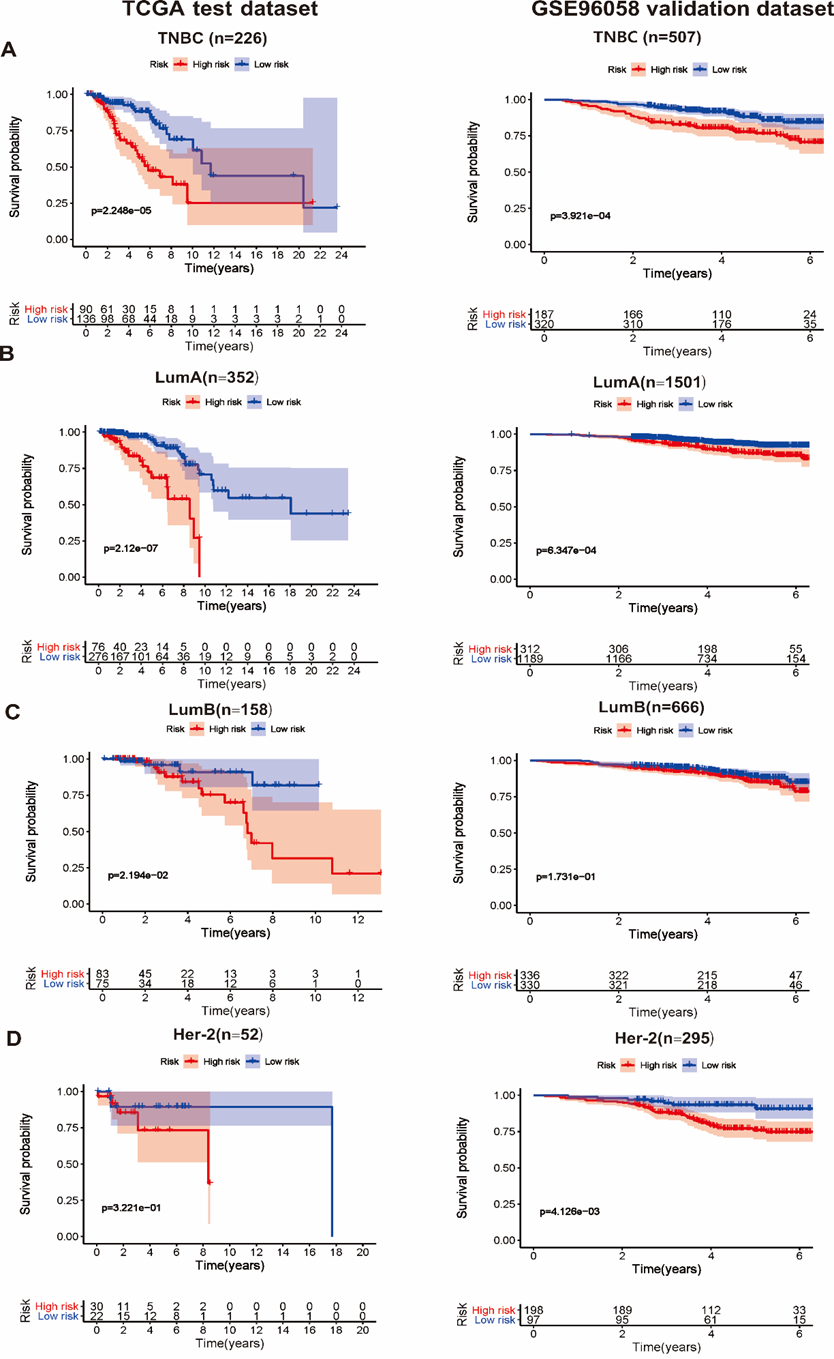


**Figure S1. Survival analysis for different molecular subtypes of breast cancer.**

(A) TNBC subtype from TCGA cohort (p=2.248e-05), GSE96058 cohort (p=3.921e-04); (B) lumA- subtype from TCGA cohort (p=2.12e-07), GSE96058 cohort (p=6.347e-04); (C) lumB- subtype from TCGA cohort (p=2.194e-02) , GSE96058 cohort (p=1.731e-01); (D) HER2- subtype from TCGA cohort(p=3.221e-01),GSE96058 cohort (p=4.126e-03). left represents TCGA cohort, right represents GSE96058 cohort.


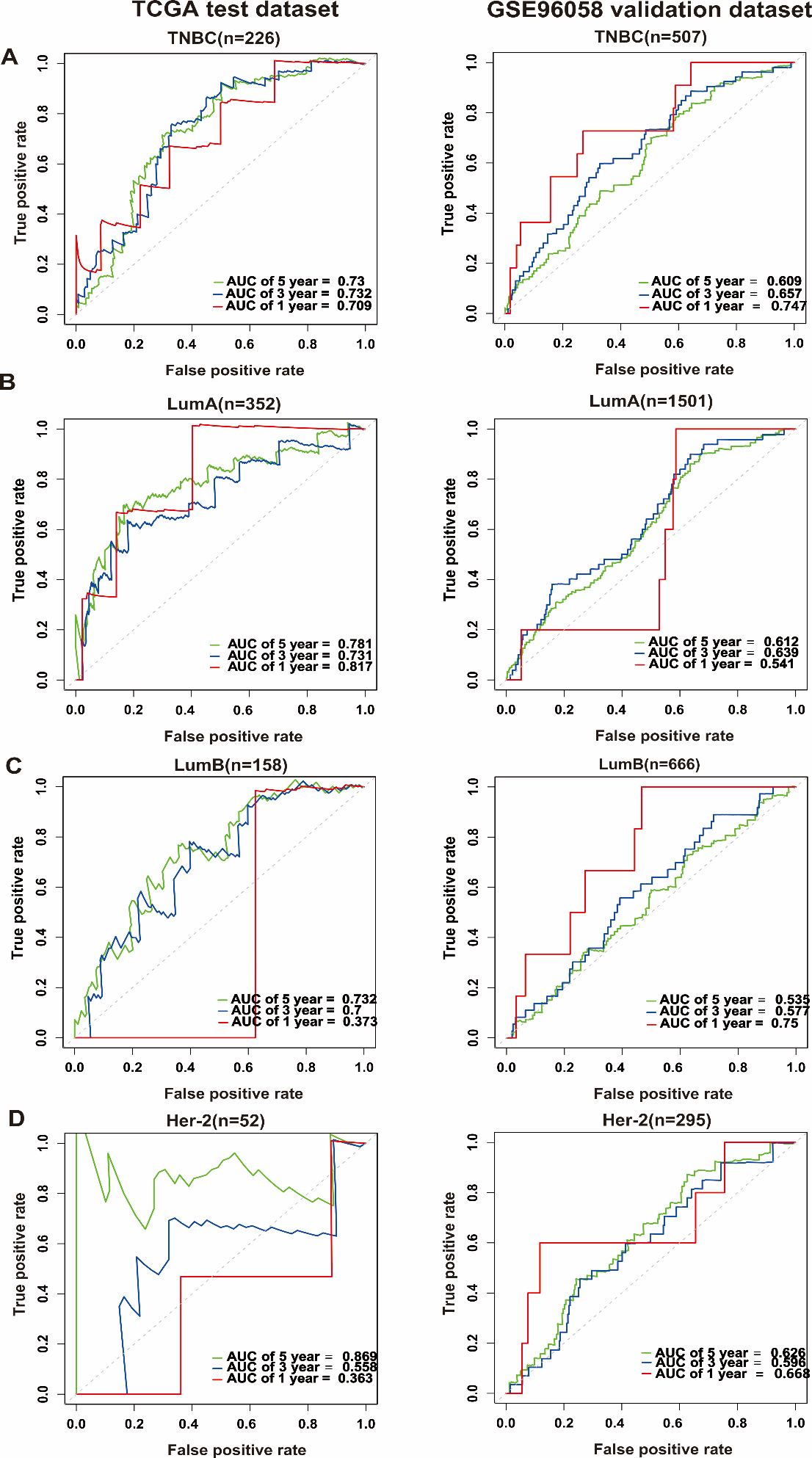


**Figure S2. Time-Dependent ROC curve analysis for different molecular subtypes of breast cancer.**

(A) TNBC subtype in TCGA cohort and GSE96058 cohort. (B) LumA- subtype in TCGA cohort and GSE96058 cohort. (C) LumB- subtype in TCGA cohort and GSE96058 cohort; (D) HER2- subtype in TCGA cohort and GSE96058 cohort. left represents TCGA cohort, right represents GSE96058 cohort.


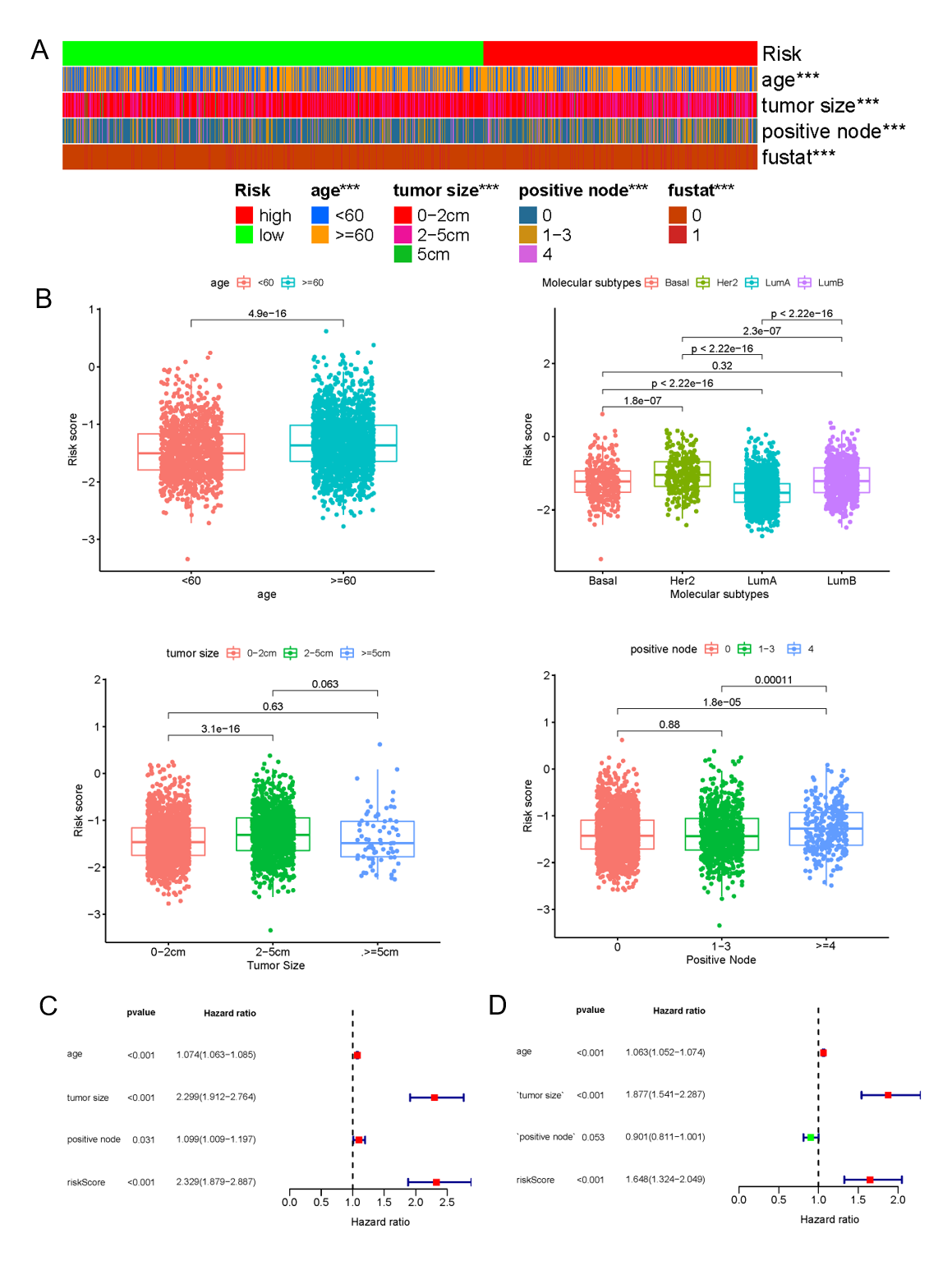


**Figure S3. Clinical evaluation of the risk model with pathological parameters in GSE96058 cohort.**

(A) The distribution of the model with the clinicopathological features including age, tumor size, positive nodes, and survival state; (B) Comparison of risk score between patients with different pathological parameters. Age, molecular subtype, tumor size, positive nodes were significantly associated with the risk score; (C) Forrest plot of univariate Cox regression analysis. The result revealed that age, tumor size, positive nodes, and risk score were statistically different. (D) Forrest plot of multivariate Cox regression analysis. Age, positive nodes, and risk score acted as independent prognostic factors.
